# Job satisfaction and organisational determinants among medical nursing assistants under hospital-based care reforms: a large cross-sectional study in China

**DOI:** 10.3389/fpubh.2026.1793432

**Published:** 2026-05-01

**Authors:** Dongmei Huang, Liyan Zhang, Shuyu Lu, Jie Qin, Caizhong Zhou, Pinyue Tao, Xiao Pan, Guining Zhang, Jinjin Wei, Caili Li, Huiqiao Huang, Xiangjie Lyu

**Affiliations:** 1 The Second Affiliated Hospital of Guangxi Medical University, Nanning, Guangxi, China; 2 Health Commission of Guangxi Zhuang Autonomous Region, Nanning, Guangxi, China; 3 The Third People’s Hospital of Nanning, Nanning, Guangxi, China; 4 The People’s Hospital of Guangxi Zhuang Autonomous Region, Nanning, Guangxi, China

**Keywords:** China, job satisfaction, medical nursing assistants, nursing assistants, training needs, work conditions

## Abstract

**Aim:**

Population ageing and increasing care demands have led to a growing reliance on nursing support personnel in hospital care systems. This study aimed to examine the working conditions, sources of stress, and job satisfaction of medical nursing assistants (MNAs), defined as non-registered nursing support personnel providing basic care and daily living assistance under the supervision of registered nurses, in China, and to identify organisational factors associated with job satisfaction.

**Design:**

Cross-sectional survey.

**Methods:**

A cross-sectional survey using a self-developed questionnaire was conducted among 6,974 MNAs working in secondary and tertiary hospitals across Guangxi Province, China, using a multi-stage sampling approach. Data collected included demographics, training experiences, work conditions, sources of stress, and job satisfaction. Job satisfaction was measured using a 5-point Likert scale (1 = very dissatisfied to 5 = very satisfied) and dichotomized as satisfied (very satisfied or satisfied) versus dissatisfied (neutral, dissatisfied, or very dissatisfied). Independent variables, such as workload, training frequency, income satisfaction, and communication quality, were operationalized as categorical variables based on self-reported responses. Chi-square tests and multivariable logistic regression were performed,with odds ratios (ORs) and 95% confidence intervals (CIs) reported to estimate the associations between independent variables and job satisfaction.

**Results:**

Most MNAs were female (92.11%) and aged 40–50 years (36.15%). Over half (53.38%) cared for ≥6 patients daily, and 39.02% frequently worked night shifts. While 61.13% perceived workload as appropriate, 20.76% reported excessive workload. Income levels were low: 48.00% earned <RMB 3,000 per month, and only 44.40% were satisfied with income. Major stressors included heavy workload (69.37%), complex interpersonal relationships (47.76%), and high care complexity (47.10%). Overall job satisfaction was 76.50%. Older age (OR = 1.179) and higher shift frequency (OR = 1.111) were associated with higher odds of job satisfaction, whereas income dissatisfaction (OR = 0.333), lack of regular training (OR = 0.618), unclear job roles (OR = 0.516), higher patient load (OR = 0.877), excessive workload (OR = 0.518), and poor communication (OR = 0.478) were associated with lower odds of job satisfaction, Nagelkerke R^2^ = 0.281, indicating moderate explanatory power(all *p* < 0.05), with income dissatisfaction showing the strongest association.

**Conclusion:**

Job satisfaction among MNAs is primarily shaped by organisational and work environment factors. Strengthening role clarity, structured training, staffing optimisation, income fairness, and communication support may enhance workforce stability and support sustainable inpatient care.

## Introduction

Population ageing is accelerating worldwide, leading to a sustained increase in demand for healthcare and long-term care services. Within this context, nursing support personnel—commonly referred to as nursing assistants, nursing aides, or healthcare support workers—have become integral to healthcare delivery systems. These workers undertake a wide range of essential tasks, including basic nursing care, assistance with activities of daily living, functional rehabilitation, and selected clinical support activities. In response to growing workforce shortages, the nursing assistant workforce has expanded substantially across countries (1–3). As health systems increasingly rely on support personnel to maintain service capacity, understanding their working conditions and occupational sustainability has become an important public health and workforce policy issue.

Although terminology and organisational arrangements vary internationally, “nursing assistant” (NA) is the most widely used umbrella term in the literature (4). Despite differences in job titles, employment models and regulatory frameworks, the core functions of NAs consistently centre on providing basic and supportive care to patients. To promote workforce standardisation and care quality, many countries have introduced formal education and training requirements, such as the U. S. Omnibus Budget Reconciliation Act (OBRA) of 1987 (5), the Care Certificate in the United Kingdom (6), and European Union–level guidelines regarding education and qualifications for nursing and care assistants (7, 8). In the Chinese context, medical nursing assistants (MNAs) are defined as non-registered nursing support personnel who provide assistance with activities of daily living, basic nursing care, and psychosocial support to hospitalised patients under the supervision of registered nurses. They do not hold formal nursing licensure and are primarily responsible for non-technical and supportive care tasks within inpatient settings. This role was formally recognised and regulated by the National Health Commission of China through policy guidance issued in 2019 (9). However, existing policies and regulations tend to focus primarily on entry requirements and training content, while comparatively limited attention has been paid to working conditions, organisational management practices and long-term professional support. Consequently, substantial variation persists in how MNAs are recruited, trained and managed across healthcare institutions (10). This imbalance between regulatory standardisation and organisational support may create structural vulnerabilities that affect job satisfaction and workforce stability.

In recent years, MNAs have played an increasingly prominent role in China’s inpatient care reforms, particularly in the implementation of “no-family-care” wards, which aim to reduce the burden on family caregivers while improving patient experience and care safety. As these initiatives expand, MNAs have become deeply embedded in the daily care of hospitalised patients. However, the MNAs workforce is predominantly employed through third-party service providers, with recruitment and training often organised by commercial agencies rather than clinical professionals. Hospitals typically have limited involvement in training design, quality control and performance evaluation, resulting in heterogeneous training quality and insufficiently standardised organisational management systems (11, 12). Such fragmented governance structures may weaken institutional accountability and blur role boundaries, potentially influencing job satisfaction and the development of professional identity.

In addition to organisational challenges, MNAs frequently face precarious and demanding working conditions, including long working hours, frequent night shifts, multiple employment arrangements and relatively low wages (13, 14). Moreover, occupational stigma and social undervaluation may undermine their professional identity, contributing to feelings of marginalisation and reduced job commitment (15, 16). While existing studies in China have primarily focused on training frameworks and management models, empirical research examining MNAs’ working conditions and job satisfaction remains limited. Several available studies have relied on relatively small samples, single-institution settings, or descriptive analyses focusing primarily on training programmes or management practices (17–19). For example, qualitative interviews and small-scale surveys have explored nursing aides’ working experiences and training needs, but these studies were typically conducted in individual institutions and lacked systematic quantitative analysis of organisational factors. Consequently, robust quantitative evidence addressing workload, work-related stress, communication experiences and job satisfaction among MNAs remains insufficient.

From an international perspective, nursing assistants are widely recognised as a workforce at risk of poor job sustainability. Studies across multiple countries report high sickness absence, low retention and substantial turnover among NAs (20–22). Job satisfaction has been consistently identified as a key determinant of workforce stability, performance and care quality. Higher job satisfaction is associated with stronger organisational commitment, lower turnover intention and improved patient outcomes, whereas dissatisfaction may contribute to burnout, absenteeism and compromised care delivery. Despite this growing body of international evidence, robust large-scale empirical studies examining job satisfaction and its determinants among MNAs in China remain scarce. Given China’s distinctive hospital-based caregiving model and the rapid expansion of “no-family-care” reforms, evidence generated from other healthcare systems may not be directly transferable to the Chinese context.

Based on existing international evidence and the Chinese policy and practice context, the present study selected key influencing factors at both the individual and organisational levels, including sociodemographic characteristics, workload and shift patterns, training experience, income satisfaction, role clarity and communication experiences. These factors have been repeatedly shown to be closely associated with job satisfaction, workforce stability and care quality among nursing assistants and other nursing support personnel (23–25). Although several Chinese studies have explored MNAs’ training systems, management models and service delivery practices (17, 26), most were conducted in single hospitals or specific care settings and primarily adopted descriptive or qualitative approaches. Few studies have applied large-scale quantitative designs capable of simultaneously examining multiple organisational and individual determinants of job satisfaction. Consequently, evidence explaining the organisational drivers of MNAs’ job satisfaction at scale remains limited in the Chinese context.

Against this background, the present study draws on a large-scale cross-sectional survey of 6,974 MNAs across secondary and tertiary hospitals in Guangxi Province. Using multivariable logistic regression, this study aims to (1) describe MNAs’ workload, sources of work-related stress, communication experiences, and overall job satisfaction, and (2) identify individual and organisational factors associated with job satisfaction.

By providing robust empirical evidence from a large provincial sample and focusing on organisational determinants, this study is among the first to quantitatively examine job satisfaction among MNAs in China. The findings are intended to inform hospital management strategies, workforce planning, and the sustainable development of inpatient caregiving services, highlighting the novel contribution of integrating organisational and individual-level predictors in a unified analytical framework.

## Methods

### Study design and participants

A cross-sectional survey was conducted between March and May 2025 among MNAs working in secondary- and tertiary-level hospitals across Guangxi Province, China. A multi-stage sampling strategy was adopted. First, a purposive sample of 20 secondary- and 15 tertiary-level hospitals from different geographic regions within Guangxi Province was selected to ensure geographic and institutional representativeness. Within each participating hospital, all eligible MNAs were invited to complete the survey (census approach within hospitals). Participants were eligible if they: (1) were formally employed as MNAs; (2) had worked in their current role for at least 3 months; and (3) voluntarily participated and provided informed consent. Individuals engaged exclusively in administrative duties or non-direct care roles were excluded from the study. A total of 7,153 MNAs were invited to participate. After data cleaning, 6,974 complete questionnaires were included in the final analysis, yielding a response rate of 97.50%.

### Survey instrument

The questionnaire was developed based on a review of relevant literature and refined through expert consultation and a pilot study. The selection of questionnaire domains was guided by existing international evidence on nursing assistants and by the Chinese policy and practice context, focusing on factors previously shown to be associated with job satisfaction, workforce stability and care quality. It comprised seven domains:

(1) sociodemographic characteristics;
(2) work characteristics, including clarity of job responsibilities, shift work patterns, number of patients cared for per day, and subjective perceptions of workload;
(3) income level and income satisfaction;
(4) scope of work activities, such as assistance with activities of daily living, basic nursing care, clinical support, psychological support, and functional rehabilitation;
(5) sources of work-related stress;
(6) communication experiences with patients and family members, as well as perceived reasons for communication difficulties; and.
(7) overall job satisfaction.

### Measurement of job satisfaction

Overall job satisfaction was measured using a single-item question on a 5-point Likert scale (1 = very dissatisfied, 5 = very satisfied). For analytical purposes, responses were dichotomized into “satisfied” (very satisfied or satisfied) and “dissatisfied” (neutral, dissatisfied, or very dissatisfied). Content validity and face validity were assessed through expert review, and the pilot survey confirmed the clarity and feasibility of the questionnaire for the target population. Internal consistency reliability of the job satisfaction scale was assessed using Cronbach’s alpha (*α* = 0.84). Exploratory factor analysis (EFA) was conducted to evaluate construct validity, with factor loadings exceeding 0.40.

### Data collection and quality control

Data were collected using an online survey platform (Wenjuanxing, China). Questionnaires were completed anonymously to ensure confidentiality. Trained investigators provided on-site guidance to facilitate questionnaire completion and to ensure data accuracy. Investigators provided procedural assistance only and did not influence participants’ responses. After data cleaning, questionnaires with missing key variables or logical inconsistencies were excluded.

### Statistical analysis

Statistical analyses were performed using SPSS version 26.0 (IBM Corp., Armonk, NY, USA). Descriptive statistics were used to summarise sociodemographic characteristics, work conditions, training experiences and sources of stress. Categorical variables were presented as frequencies and percentages, while continuous variables were described using means and standard deviations. Univariate analyses were conducted to examine associations between job satisfaction and participant characteristics using chi-square tests or independent-samples t tests, as appropriate. Variables showing statistical significance in univariate analyses were subsequently entered into multivariable logistic regression models to identify independent factors associated with job satisfaction.

In the logistic regression model, categorical variables were entered using dummy coding, with the lowest category as the reference. Age (years), shift frequency (daily, frequent, occasional, never), and patient load (1, 2–3, 4–5, ≥6) were treated as categorical variables. Ordinal variables were not treated as continuous. Multicollinearity was assessed using variance inflation factors (VIFs), and all VIF values were below 5, indicating no significant multicollinearity. Model fit was evaluated using the Hosmer–Lemeshow goodness-of-fit test, and the linearity of the logit for continuous predictors was checked to ensure logistic regression assumptions were met. Odds ratios (ORs) with 95% confidence intervals (CIs) were reported. This study was reported in accordance with the Strengthening the Reporting of Observational Studies in Epidemiology (STROBE) guidelines.

### Ethics approval and consent to participate

The study protocol was reviewed and approved by the Ethics Committee of the Second Affiliated Hospital of Guangxi Medical University. All participants provided written informed consent prior to enrolment. Before signing, each participant received a detailed explanation of the study objectives, procedures, potential risks, and benefits, and was given sufficient time to consider participation and ask questions. Participation was entirely voluntary. The study was conducted in accordance with the ethical principles of the Declaration of Helsinki.

## Results

### Demographic characteristics and training status of MNAs

The majority of participants were female (92.11%), with ages mainly concentrated between 30 and 50 years. Approximately one-third had more than 8 years of work experience, while one-quarter had been employed for 1–3 years, indicating a mixed workforce of experienced and newly employed assistants. Nearly half were hospital-employed, and a comparable proportion were agency-dispatched.

Over 90% had received some form of professional training and almost all expressed a need for training, but only about one-third reported high training frequency, suggesting insufficient training intensity (Table 1).

**Table 1:** Sociodemographic characteristics and training experiences of MNAs (*n* = 6,974).

Variable	Category	*n*	%
Gender	Male/female	550/6,424	7.89/92.11
Age (years)	<20 / 20–30 / 30–40 / 40–50 / >50	75/1069/1784/2521/1525	1.08/15.33/25.58/36.15/21.87
Work experience (years)	<1 / 1–3 / 3–5 / 5–8 / >8	983/1790/1165/925/2111	14.09/25.67/16.70/13.26/30.27
Employment type	Hospital-employed/labor company/patient- or family-hired	3242/3106/626	46.49/44.54/8.98
Education	No nursing-related education/secondary nursing/college degree or higher	3129/1548/2297	44.87/22.20/32.94
Training need	Strong need/need/no need	3598/3233/143	51.59/46.36/2.05
Training experience	Received professional training/not received	6280/694	90.03/9.97
Training frequency	Very high/moderate/occasionally/rarely/never	2451/3474/788/180/81	35.14/49.81/11.31/2.58/1.16

### Work conditions, stressors, and job satisfaction

Most participants reported clear job responsibilities (85.9%). Night shifts were common (39.0% frequent, 15.7% daily), and patient load varied, with 53.4% caring for ≥6 patients. Workload was deemed appropriate by 61.1%, excessive by 20.8%.

Income was low: 48.0% earned <3,000 CNY/month, 41.5% earned 3,000–5,000 CNY; only 44.4% were satisfied. Communication with patients/families was generally smooth (74.4%), and the overall job satisfaction score was 3.78 ± 0.72, with 76.5% of participants reporting satisfaction (very satisfied or satisfied). Work focused on activities of daily living (84.1%) and basic care (76.2%), with less involvement in psychological support (59.0%), clinical care (54.8%), or functional training (46.7%). Major stressors included high workload (69.4%), complex interpersonal relationships (47.8%), care difficulty (47.1%), limited promotion (38.8%), and lack of professional training (34.1%). Poor communication stemmed from patients/families being unappreciative (73.5%), lacking trust (63.3%), or misunderstanding service content (49.4%) (Table 2).

**Table 2:** Work conditions, workload, stressors, and job satisfaction of MNAs (*n* = 6,974).

Variable	Category	*n*	%
Clarity of job responsibilities	Very clear/not clear/none	5987/790/197	85.85/11.33/2.82
Shift work frequency	Daily/frequently/occasionally/never	1096/2721/1887/1270	15.72/39.02/27.06/18.21
Patients per day	1/2–3/4–5/≥6	1393/1111/747/3723	19.97/15.93/10.71/53.38
Perceived workload	Very appropriate/appropriate/excessive/low	1176/4263/1448/87	16.86/61.13/20.76/1.25
Monthly income (CNY)	7,000	3348/2896/610/120	48/41.53/8.75/1.72
Income satisfaction	Very satisfied/satisfied/neutral/dissatisfied/very dissatisfied	995/2101/2498/1057/323	14.27/30.13/35.82/15.16/4.63
Communication with patients	Smooth/Moderate/Poor	5191/1734/49	74.43/24.86/0.70
Overall job satisfaction	Satisfied/dissatisfied	5335/1639	76.50/23.50
Work content (multiple responses)	ADL care (e.g., hygiene, feeding, sleep, toileting)/basic nursing care (e.g., observation, cleaning, pressure injury prevention, mobilization)/clinical care (e.g., specimen collection, medication administration, emergency care)/Psychological support (e.g., comforting, end-of-life care)/Functional exercise	5864/5314/3823/4114/3256	84.08/76.20/54.82/58.99/46.69
Sources of work stress (multiple responses)	High workload/lack of professional training/limited career advancement/high nursing difficulty/complex interpersonal relationships	4838/2381/2708/3285/3331	69.37/34.14/38.83/47.10/47.76
Reasons for poor communication (multiple responses)	Patients/families’ lack of understanding/lack of trust/unclear about service content/standards/perceived unreasonable fees/limited own communication skills	5126/4412/3445/3232/2294	73.50/63.26/49.40/46.34/32.89

### Univariate analysis of job satisfaction

Job satisfaction (satisfied = 1; dissatisfied = 0) was analyzed using χ^2^ tests. Factors significantly associated with job satisfaction included age, employment type, educational background, training needs, training experience, monthly income, income satisfaction, clarity of job responsibilities, number of patients cared for, perceived workload, shift work frequency, and communication with patients (all *p* < 0.05) (Table 3).

**Table 3:** Univariate analysis of factors associated with overall job satisfaction (*n* = 6,974).

Variable	Category	Satisfied n (%)	Dissatisfied *n* (%)	*χ* ^2^	*p*
Gender	Male	428 (77.8)	122 (22.2)	0.578	0.464
	Female	4,907 (76.4)	1,517 (23.6)		
Age (years)	<20	48 (64.0)	27 (36.0)	75.52	<0.001
	20–30	793 (74.2)	276 (25.8)		
	30–40	1,349 (75.6)	435 (24.4)		
	40–50	1857 (73.7)	664 (26.3)		
	>50	1,288 (84.5)	237 (15.5)		
Work experience (years)	<1	738 (75.1)	245 (24.9)	5.96	0.202
	1–3	1,343 (75.0)	447 (25.0)		
	3–5	909 (78.0)	256 (22.0)		
	5–8	709 (76.6)	216 (23.4)		
	>8	1,636 (77.5)	475 (22.5)		
Employment type	Hospital-employed	2,461 (75.9)	781 (24.1)	15.72	<0.001
	Agency-dispatched	2,355 (75.8)	751 (24.2)		
	Patient/family-employed	519 (82.9)	107 (17.1)		
Education	No nursing-related education	2,333 (74.6)	796 (25.4)	15.43	<0.001
	Secondary nursing	1,233 (79.6)	315 (20.4)		
	College degree or above	1769 (77.0)	528 (23.0)		
Training needs	Very needed	2,944 (81.8)	654 (18.2)	127.98	<0.001
	Needed	2,306 (71.3)	927 (28.7)		
	Not needed	85 (59.4)	58 (40.6)		
Training experience	Yes	5,874 (93.5)	406 (6.5)	43.49	<0.001
	No	461 (66.4)	233 (33.6)		
Monthly income (CNY)	<3,000	2,152 (87.8)	299 (12.2)	518.72	<0.001
	3,000–5,000	2,625 (75.6)	849 (24.4)		
	5,001–7,000	412 (52.3)	376 (47.7)		
	>7,000	85 (47.2)	95 (52.8)		
Income satisfaction	Satisfied	4,717 (89.2)	571 (10.8)	187.38	<0.001
	Dissatisfied	618 (36.6)	1,068 (63.4)		
Clarity of job responsibilities	Very clear	981 (98.6)	14 (1.4)	1656.59	<0.001
	Clear	2048 (97.5)	53 (2.5)		
	Unclear	1,678 (67.2)	820 (32.8)		
	Very unclear	506 (47.9)	551 (52.1)		
	None	122 (37.8)	201 (62.2)		
Patients per day	1	4,855 (91.8)	430 (8.2)	517.24	<0.001
	2–3	360 (82.4)	77 (17.6)		
	≥4	1,132 (90.4)	120 (9.6)		
Workload perception	Appropriate	5,455 (88.0)	399 (12.0)	145.87	<0.001
	Excessive/Low	880 (42.3)	1,200 (57.7)		
Shift work frequency	Daily	1,127 (95.8)	49 (4.2)	1012.35	<0.001
	Frequent	3,472 (81.5)	791 (18.5)		
	Occasional	680 (46.9)	768 (53.1)		
	Never	56 (64.4)	31 (35.6)		
Communication with patients	Smooth	3,922 (82.2)	847 (17.8)	47.80	<0.001
	Moderate/Poor	1,413 (63.9)	792 (36.1)		

### Multivariate analysis of job satisfaction

Variables significant in the univariate analysis were entered into a multivariate logistic regression model. Older age (OR = 1.179, 95% CI: 1.087–1.278) and higher frequency of shift work (OR = 1.111, 95% CI: 1.029–1.201) were positive predictors of job satisfaction. Interestingly, higher shift frequency may reflect contextual factors such as shift-related allowances, schedule predictability, or self-selection of workers who tolerate shift work. Negative predictors included income dissatisfaction (OR = 0.333, 95% CI: 0.304–0.364), lack of regular professional training (OR = 0.618, 95% CI: 0.562–0.681), unclear job responsibilities (OR = 0.516, 95% CI: 0.444–0.601), higher patient load (OR = 0.877, 95% CI: 0.819–0.940), perceived excessive workload (OR = 0.518, 95% CI: 0.458–0.586), and poor communication with patients(OR = 0.478, 95% CI: 0.417–0.549). Income satisfaction was coded as dissatisfaction versus satisfaction (neutral, dissatisfied, or very dissatisfied vs. satisfied or very satisfied). The model explained 28.1% of the variance (Nagelkerke R^2^ = 0.281) (Table 4).

**Table 4:** Multivariate logistic regression analysis of factors associated with overall job satisfaction (*n* = 6,974).

Variable	*β*	*SE*	Wald	*p*	OR	95% CI
Age	0.164	0.041	15.857	<0.001	1.179	1.087–1.278
Employment type	0.066	0.063	1.113	0.291	1.069	0.945–1.209
Education	0.090	0.049	3.341	0.068	1.095	0.993–1.206
Training needs	−0.169	0.066	6.575	0.010	0.844	0.742–0.961
Monthly income	−0.063	0.059	1.127	0.288	0.939	0.837–1.054
Income satisfaction	−1.100	0.045	587.264	<0.001	0.333	0.304–0.364
Training experience	−0.481	0.049	95.929	<0.001	0.618	0.562–0.681
Clarity of job responsibilities	−0.661	0.077	73.452	<0.001	0.516	0.444–0.601
Patients per day	−0.131	0.035	13.943	<0.001	0.877	0.819–0.940
Perceived workload	−0.658	0.063	108.238	<0.001	0.518	0.458–0.586
Shift work frequency	0.106	0.040	7.143	0.008	1.111	1.029–1.201
Communication with patients	−0.737	0.070	111.045	<0.001	0.478	0.417–0.549

Job satisfaction was dichotomized as satisfied (very satisfied or satisfied) vs. dissatisfied (neutral, dissatisfied, or very dissatisfied). For multivariate logistic regression, the first category of each variable was used as the reference group (e.g., daily shift work for shift frequency, satisfied/very satisfied for income satisfaction, lowest patient load for number of patients).

## Discussion

This large-scale cross-sectional study comprehensively examines the working conditions, training experiences, sources of stress, and job satisfaction of MNAs in China. Our findings reveal a workforce under high care demands, substantial workload, and strong training needs, with limited organizational support.

To our knowledge, this is one of the largest quantitative studies to date examining organisational determinants of job satisfaction among MNAs in China using multivariable modelling. These results provide robust empirical evidence to inform hospital-based care reform and workforce policy development. This is particularly relevant in China, where the rapid expansion of hospital-based caregiving services and the ongoing reform of non-family caregiving models have substantially increased the demand for MNAs within inpatient care systems.

The demographic profile indicates that MNAs are predominantly middle-aged and older women, with most participants aged 30–50 years and a considerable proportion with long work tenure. Approximately one-quarter of respondents had less than 3 years of work experience, suggesting ongoing turnover and persistent challenges related to job attractiveness and occupational sustainability (25, 27–28). This highlights the need for targeted strategies to enhance retention and career continuity among MNAs. Training is widespread but often insufficient. Although over 90% had received some professional training and nearly all expressed a need for further training, only one-third reported high training frequency. Insufficient training may compromise role competence, safety awareness, and professional confidence, particularly as non-family caregiving models expand and inpatient care expectations rise.

Workload and remuneration remain key challenges. More than half of the MNAs cared for six or more patients per day, and a substantial proportion engaged in frequent or daily night or shift work, indicating a generally high work intensity. Most respondents rated their workload as appropriate; however, about one-fifth perceived it as excessive, possibly reflecting constrained employment alternatives or adjusted occupational expectations. Nearly half of the respondents earned less than 3,000 RMB per month, and almost 90% reported monthly incomes below 5,000 RMB, accompanied by low income satisfaction. Lower income satisfaction was associated with lower odds of job satisfaction (OR = 0.333), highlighting the role of perceived distributive fairness in shaping occupational attitudes (25, 29–30).

The overall job satisfaction score (mean = 3.78 ± 0.72) indicated that 76.50% of participants were classified as satisfied (very satisfied or satisfied), suggesting a moderate-to-high level of satisfaction. This level exceeds that reported among nursing assistants in some developing countries but remains lower than levels observed among registered nurses in high-income settings (31, 32). Differences in resource allocation, labor protection, and professional support systems may partially explain this disparity. Previous research has suggested that older age and longer work experience may partly explain this relatively favorable satisfaction (29). In addition, cultural and labour-market factors may also contribute to this pattern. For many middle-aged women entering the MNAs workforce in China, the job may represent relatively stable employment compared with alternative low-skilled occupations. Consequently, job satisfaction may reflect broader perceptions of employment stability, social participation, and meaningful engagement in healthcare services rather than salary alone.

Importantly, multivariate analysis demonstrated that unclear job roles, lack of regular training, higher patient loads, perceived excessive workload, and poor communication were associated with lower odds of being satisfied with the job, rather than implying causal effects. These determinants are largely organizational and environmental rather than individual in nature. This finding reinforces the theoretical perspective that job satisfaction among support healthcare workers is shaped more strongly by organisational structures and managerial practices than by immutable individual characteristics. Insufficient training may constrain perceived competence and career prospects (33), while excessive patient numbers and workload directly increase physical and psychological strain (34–36). Communication difficulties further intensify emotional labor and interpersonal stress (37). Among these factors, role clarity exerted a particularly strong association with job satisfaction (38, 39), and dissatisfaction with promotion and training opportunities may outweigh the effects of workload or pay alone (40). Establishing standardized job descriptions and tiered training systems may therefore improve satisfaction and interprofessional collaboration.

Interestingly, both age and shift work were associated with higher odds of being satisfied with the job. This finding aligns with the results reported by Stone et al. (41) and is consistent with several empirical studies reporting that shift work does not necessarily reduce job satisfaction among healthcare workers when organisational support and scheduling flexibility are available (42–44). However, it contrasts with studies identifying negative associations between shift work and satisfaction that attribute dissatisfaction primarily to circadian rhythm disruption, fatigue and work–life imbalance (45, 46). Such inconsistencies suggest that contextual and population-specific factors may shape the relationship between shift work and job satisfaction and may reflect an “adaptation–selection” mechanism. Older workers may develop more stable occupational expectations and stronger coping capacities over time, resulting in greater tolerance of work-related stressors. Similarly, individuals who remain in shift or night work may self-select based on physical adaptability, time management skills, or personal preferences. Previous research has also shown that financial incentives, predictable scheduling, and perceived organisational support may mitigate the negative effects of shift work and contribute to higher job satisfaction among healthcare personnel (47). In some institutions, shift work may also be accompanied by financial allowances, predictable schedules, or perceived job security, partially offsetting its physiological burden. Previous studies have highlighted the importance of work experience, occupational adaptability, incentives, and scheduling autonomy in shaping job satisfaction and retention (48–51). Within the Chinese hospital context, shift work may also be linked to institutional employment arrangements and eligibility for shift allowances, and employees who accept shift schedules may perceive greater job stability or organizational integration. The positive association observed in this study may therefore reflect contextual factors specific to Chinese hospital-based employment models, including shift-related allowances, relative job security, or informal scheduling flexibility. These findings suggest that, when workload is appropriately managed, optimizing shift systems and enhancing scheduling flexibility may contribute positively to job satisfaction.

Communication experiences emerged as another critical yet frequently overlooked determinant. Notably, communication factors remained significant in multivariable analysis, with poor communication associated with lower odds of job satisfaction (OR = 0.478), rather than causing dissatisfaction. Barriers were primarily related to patients’ and families’ lack of understanding, trust deficits, and limited awareness of service scope and standards. These findings indicate that MNAs engage not only in physical care but also in substantial emotional and relational labor. Prior research has demonstrated that perceived communication quality is closely associated with job satisfaction and organizational commitment among nursing assistants, whereas poor communication increases dissatisfaction and turnover intention (52). Furthermore, clear communication and role recognition from supervisors and colleagues have been identified as important environmental contributors to job satisfaction (53). In the absence of clear professional identity and institutional support, negative communication experiences may intensify feelings of marginalization and occupational stigma, thereby undermining professional identity and work engagement. Improving communication among MNAs, patients, families, and healthcare teams is therefore not only a service quality issue but also a key mechanism influencing occupational identity and organizational embeddedness. Integrating communication training into standardized MNA training curricula may therefore represent a feasible intervention pathway.

### What this study adds

This study extends existing literature in three important ways. First, it provides large-scale quantitative evidence from China examining organisational determinants of MNA job satisfaction using multivariable modelling. Second, it demonstrates that structural and managerial factors—particularly role clarity, training regularity, workload, and communication—exert stronger influence than most individual demographic characteristics. Third, it highlights the central role of perceived income fairness and organisational support within the context of expanding hospital-based caregiving reforms in China. These findings contribute to the international understanding of support healthcare workforce sustainability in rapidly evolving health systems.

### Theoretical and policy implications

From a theoretical perspective, the findings support organisational and work environment models of job satisfaction, suggesting that structural and managerial factors—such as role clarity, training systems, and workload allocation—may exert stronger influence on job satisfaction than most individual demographic characteristics. From a policy perspective, the results highlight the need for more standardized employment models, training frameworks, and regulatory mechanisms for MNAs within China’s rapidly evolving hospital-based care system. Strengthening institutional governance and professional recognition of MNAs may contribute to improving workforce stability and care quality.

### Strengths and limitations

This study has several strengths. It was based on a large sample of MNAs from secondary and tertiary hospitals, enhancing the robustness of the findings. In addition, multiple organizational and work-related factors were examined simultaneously, allowing a comprehensive assessment of determinants of job satisfaction. Several limitations should be acknowledged. First, the cross-sectional design limits causal interpretation of the observed associations. Second, data were self-reported and may be affected by response bias. Third, although the questionnaire was developed based on literature review and expert consultation, it was self-developed rather than based on a previously validated international instrument, which may limit comparability across studies despite acceptable reliability indicators. In addition, the use of a self-developed instrument may introduce potential measurement limitations, such as incomplete capture of certain constructs related to job satisfaction or organisational experiences. As a result, direct comparison with findings derived from widely used international scales may be limited. Future studies could further validate and standardise the measurement tool or adopt internationally recognised instruments to enhance cross-study comparability. Fourth, although the sample size was large, the study was conducted in a single province, which may limit generalizability to other regions. Fifth, online data collection may introduce potential selection bias, as MNAs with limited digital access or lower engagement may have been underrepresented. Future longitudinal and multi-site studies are needed to confirm these findings.

### Implications for nursing management

The findings highlight several actionable priorities for nursing management. Clear role definition and standardized job descriptions are essential to reduce role ambiguity and improve job satisfaction among MNAs. At the policy level, establishing province-wide or national standards for MNA job descriptions and competency frameworks may reduce inter-institutional variability and enhance workforce professionalisation. Continuous and structured training programs, rather than sporadic training, should be strengthened to enhance competence and professional confidence. Linking training completion to tiered certification or career progression pathways may further strengthen retention. Workload and staffing allocation require careful management, as high patient loads and perceived excessive workload were strongly associated with lower job satisfaction. Developing evidence-based staffing ratios for MNAs in hospital settings could represent a critical reform direction. Nursing managers should consider optimizing staffing models and shift arrangements. In addition, although salary policies are often institutionally determined, improving perceived income fairness through transparent compensation mechanisms and shift-related incentives may enhance satisfaction. Finally, communication should be recognized as a managerial issue. Embedding structured communication protocols and conflict mediation mechanisms within hospital management systems may mitigate relational stress and improve organisational climate.

From a lifecycle perspective, workforce management strategies should be tailored to different career stages of MNAs. For newly recruited MNAs, structured onboarding and basic skills training are essential to facilitate role adaptation. For mid-career MNAs, continuous professional development opportunities and supportive supervision may enhance job competence and retention. For more experienced MNAs, the development of clear career pathways, role advancement opportunities, and recognition mechanisms may help strengthen professional identity and long-term workforce stability. Integrating such stage-specific strategies may contribute to a more sustainable and resilient MNA workforce.

## Conclusion

Based on a large-scale cross-sectional survey, this study examined the working conditions and job satisfaction of MNAs in China. Overall job satisfaction was moderate to high; however, notable gaps remain in income adequacy, training support, role clarity, workload, and communication. Multivariate analysis showed that job satisfaction was largely influenced by organizational and work environment factors, with income satisfaction, training adequacy, role clarity, workload, and communication quality emerging as key determinants.

These findings highlight the need for system-level management strategies, including clarifying job roles, enhancing training and career development pathways, optimizing staffing allocation, and fostering supportive communication environments. Given the ongoing expansion of hospital-based caregiving and “no-family caregiver” service models in China, strengthening organizational support mechanisms for MNAs is essential to ensure workforce sustainability. Strengthening these areas may improve job satisfaction and workforce stability, thereby supporting the quality and sustainability of inpatient care.

However, due to the cross-sectional design, causal relationships cannot be inferred, and self-reported data may be subject to response bias. These limitations suggest that the observed associations should be interpreted with caution, and the effectiveness of management or policy interventions cannot be directly established from this study. Future longitudinal and multi-regional studies are warranted to further explore temporal dynamics, causal relationships, and policy impacts.

From a practical perspective, hospital administrators and policymakers should prioritise the development of standardized competency frameworks, transparent compensation systems, and structured training pathways to enhance the professionalization and retention of MNAs.

## Data Availability

The original contributions presented in the study are included in the article/supplementary material, further inquiries can be directed to the corresponding authors.
